# Glycolysis is suppressed by DCZ0801-induced inactivation of the Akt/mTOR pathway in Multiple Myeloma

**DOI:** 10.7150/jca.45146

**Published:** 2020-06-15

**Authors:** Qilin Feng, Qingchun Yao, Bo Li, Yongsheng Xie, Hui Zhang, Zhijian Xu, Kang Lu, Ke Hu, Yao Cheng, Bingqing Shi, Cheng Huang, Liping Li, Xiaosong Wu, Shanxi You, Jumei Shi, Weiliang Zhu

**Affiliations:** 1 Department of Hematology, Shanghai Tenth People's Hospital, Tongji University School of Medicine, Shanghai 200072, China.; 2 Department of Oncology, Taizhou Fourth People's Hospital, Jiangsu 225300, China.; 3 CAS Key Laboratory of Receptor Research; Drug Discovery and Design Center, Shanghai Institute of Materia Medica, Chinese Academy of Sciences, Shanghai 201203, China.

**Keywords:** DCZ0801, multiple myeloma, apoptosis, cell cycle, glycolysis

## Abstract

Multiple myeloma (MM) is a highly invasive and incurable plasma cell malignant disease with frequent recurrence. DCZ0801 is a natural compound synthesized from osalmide and pterostilbene and has few adverse effects. Here, we aimed to observe the therapeutic effects of DCZ0801 on myeloma cells and clarify the specific molecular mechanism underlying its anti-tumor activity. The Cell Counting Kit-8 assay, apoptosis detection, cell cycle analysis, western blot analysis, and tumor xenograft models were used to determine the effect of DCZ0801 treatment both *in vivo* and *in vitro*. We revealed that DCZ0801 treatment suppressed MM cell survival by inducing apoptosis and blocking the cell cycle at S phase. Deranged glycolysis and downregulated Akt/mTOR pathway may also be responsible for cell proliferation inhibition. Moreover, DCZ0801 treatment could remarkably reduce the tumor size in the xenograft mouse model. Therefore these findings indicate that DCZ0801 can be used as a novel therapeutic drug for patients suffering from multiple myeloma.

## Introduction

Multiple myeloma is the second most common hematological malignancy described as an uncontrolled growth of monoclonal plasma cells 1. The annual incidence of multiple myeloma worldwide is 6-7/100,000, accounting for 1.8% of all new cancer cases in the USA. In 2019, reports estimated that there would be 32,110 new cases of myeloma and that nearly 12,960 people would die of this disease. Myeloma is more common among elderly people, aged 65-74 years. The percent 5-year survival is 52.5% 2,3. Due to advances in targeted drugs and new technologies such as immunomodulators, proteasome inhibitors, monoclonal antibodies, deacetylase inhibitors, and CAR-T cell therapy for the treatment of multiple myeloma, the progression-free survival and overall survival rate of patients with myeloma are prolonged and improved 4,5. However, drug resistance is ultimately inevitable and the disease becomes more aggressive after relapse; the subsequent recurrence time of the disease is also shortened 6. Therefore, myeloma remains incurable and prone to relapse. Thus, there is an urgent need to explore new molecular targeted drugs for the treatment of myeloma.

Metabolism is a complex biochemical process that is essential to maintain normal cell survival and growth, including the synthesis and catabolism of glucose, fatty acids, and amino acids 7. Among these, glucose is the main energy source for maintaining normal human activities. In aerobic conditions, glucose metabolism produces pyruvate, and eventually oxidizes into water and carbon dioxide in the mitochondria. Under anaerobic conditions, glucose is eventually converted into lactic acid 8,9. Nobel Prize winner Otto Warburg found that cancer cells prefer anaerobic respiration even in the presence of sufficient oxygen supply, which increases glucose consumption and subsequent lactate production in cancer cells. This phenomenon is famously known as the Warburg effect or aerobic glycolysis 10-12. Glucose transporter-1 (GLUT-1) is a member of the glucose transporter family. In tumor cells with enhanced glycolysis, the expression levels of GLUT1 increase, which accelerates the uptake of glucose and quickly gains energy required for cell proliferation 13. Pyruvate kinase (PK) is a rate-limiting terminal glycolytic enzyme that has an essential role in the aerobic glycolysis of cancer cells, converting phosphoenolpyruvate (PEP) to pyruvate and ADP to ATP in the cytoplasm 14,15. There are four isoforms of PK, namely PKM1, PKM2, PKL, and PKR. Of these, PKM2 is highly expressed in various tumor tissues, supporting the growth of cancer cells and stimulating tumor proliferation 16-18. Extracellular signal regulated kinase1/2 (ERK1/2) binds and phosphorylates the Ser37 site of PKM2 which further induces the expression of PKM2 in the form of positive feedback. Simultaneously, PKM2 acts as a transcriptional coactivator of hypoxia inducible factor 1 - α (HIF1 α) and upregulates its expression, thus increasing the expression of MCT4 and sensitizing the glycolytic activity of tumor cells 19-21. The lactate dehydrogenase A (LDHA ) is the final enzyme of aerobic glycolysis, which converts pyruvate and NADH to lactate and NAD. Studies documented that inhibition of LDHA restrains glycolysis essential to tumor maintenance and progression 22. Lactate metabolism is also involved in carcinogenesis and increases with tumor growth 23. Monocarboxylate transporters 4 (MCT4) acts as intracellular lactate exporters which plays an important role in glycolysis activity 24. Moreover high expression of MCT4 correlates with poor tumor prognosis 25.

Mammalian/mechanistic target of rapamycin (mTOR) is a serine/threonine kinase with a molecular weight of 289 kDa. In many malignant tumors, mTOR is abnormally activated and plays an important role in cell differentiation, proliferation and survival including prostate cancer, ovarian cancer and multiple myeloma 26-28. Protein kinase B (Akt), a upstream of mTOR, activates mTOR by phosphorylating the TSC1/TSC2 complex 29. Simultaneously mTOR also mediates the phosphorylation of Akt on Ser473 to regulate mRNA translation and cell survival 30. Ribosomal protein S6 kinase (p70S6K) and eukaryotic initiation factor 4E binding protein (4E-BP1), downstream effectors of mTOR, are known to regulate protein translation and biosynthesis of lipids and nucleotides, thus endowing cancer cells with the ability of growth and survival 31. Further, mTOR pathway activation influences the expression of glycolytic enzymes to induce an increase in glycolysis, eventually promoting tumor cell growth 32. In multiple myeloma, mTOR knock-down inhibited the proliferation of MM cells and rapamycin, which is an inhibitor of mTOR, has been reported to suppress the rate of glycolysis 33,34.

DCZ0801 is a new natural product of the combination of osalmide and pterostilbene. The present study examined the potential anti-myeloma effects of DCZ0801, both *in vivo* and *in vitro*, and investigated the mechanisms underlying its effects.

## Materials and Methods

### Cell lines and cell culture

Human myeloma cell lines RPMI-8226, U266 and OPM2 were purchased from the American Type Culture Collection (Manassas, VA, USA); OCI-MY5 and the bortezomib-resistant cell line RPMI-8226/R5 were kindly provided by Fenghuang Zhan (Department of Internal Medicine, University of Iowa, Iowa City, USA). Primary cells are CD138^+^ mononuclear cells isolated from the bone marrow of patients with multiple myeloma by Ficoll-Hypaque density gradient centrifugation. Written informed consent was signed by multiple myeloma patients in accordance with the Helsinki Declaration. The institutional review board of the Shanghai Tenth People's Hospital, Tongji University approved this protocol for collection of clinical samples. Human myeloma cell lines and CD138^+^ myeloma cells were cultured in RPMI-1640 (Gibco, Carlsbad, CA, USA) containing 10% fetal bovine serum (FBS; Gibco, BRL, USA) and 1% penicillin-streptomycin (PS; Gibco, Carlsbad, CA, USA). All cells were incubated in artificial incubator containing 5% CO_2_ at 37°C.

### Reagents

DCZ0801 was synthesized by the Shanghai Institute of Materia Medica (Chinese Academy of Sciences, Shanghai, China). It was dissolved in dimethyl-sulfoxide (DMSO; Sigma-Aldrich, St. Louis, MO, USA) at a concentration of 100 mM, stored at -20°C and diluted at desired concentrations into each well plate with cell suspension. The Cell Counting Kit-8 (CCK-8) was obtained from Yeasen (Shanghai, China), the Annexin-V/propidium iodide (PI) apoptosis detection kit was purchased from BD Pharmingen (Franklin Lakes, USA), and Z-VAD-FMK was provided by Selleck Chemicals (Houston, USA). Antibodies against cleaved caspase-8 (#9496), caspase-9 (#9508), caspase-3 (#9662), CDK2 (#2546), cdc25A (#3652), cyclinA2 (#1547), Akt (#9272), p-Akt (Ser473, #9271), p-4E-BP1 (Ser65, #9456), 4E-BP1 (#9644), p-mTOR (Ser2481, #2974), mTOR (#2983), p-p70S6K (Thr389, #9205), p70S6K (#2708), p-ERK1/2 (Ser383, #9181), STAT3 (#9139), PKM2 (#4053) and β-actin (#3700) were purchased from Cell Signaling Technology (Beverly, USA). Antibodies against poly ADP-ribose polymerase (PARP) (#ab74290), p-STAT3 (Y705, #ab76315), ERK1/2 (#ab17942), Anti-Glucose Transporter 1 (GLUT-1) (#ab115730) and Anti-Lactate Dehydrogenase (#ab52488) were from Abcam (Cambridge, UK). MCT4 Antibody (#22787-1-AP) was obtained from proteintech (Chicago, USA).

### Cell viability experiment

Human myeloma cell lines treated with DCZ0801 (15, 30, 60, 90, 120, or 180 μM) were cultured in 96-well plates at a density of 20 x 10^4^ cells/ml. After 48 h, Cell Counting Kit-8 (CCK-8) colorimetric assay (Yeasen Biotechnology Co., Ltd, Shanghai, China) was used to measure viable cells exposed to different concentrations of DCZ0801. The half maximal inhibitory concentration (IC50) value and the combination index (CI) were calculated using the CalcuSyn software version.

### Apoptosis detection

Myeloma cells are cultured in a 24-well plate at a density of 20 × 10^4^ cells/ml, simultaneously, different concentrations of DCZ0801 (0, 60, 120 μM) and/or Z-VAD-FMK (50 μM) were added to each well for 48 h. Cell suspension is collected and supernatant is decanted after centrifugation at 142 × g for 5 min. Double staining was performed using the BD Pharmingen TM Annexin V/PI Apoptosis Detection Kit, followed by detection of apoptosis by BD FASC Canto II flow cytometry (BD Biosciences, San Jose, CA, USA). Annexin V^+^/PI^-^ (early apoptosis) and Annexin V^+^/PI^+^ (late apoptosis) were identified as apoptotic cells.

### Cell cycle analysis

Multiple myeloma cells supplemented with (0, 60, 120 μM) DCZ0801 were cultured in 24-wells for 24 h at a density of 20 × 10^4^ cells/ml. Subsequently, cells was washed with PBS and fixed with pre-cooled 70% ethanol at -20°C overnight. Cells fixed by ethanol were again washed by PBS and incubated with 300 μL PI/RNase staining buffer (BD Pharmingen Franklin Lakes, NJ, USA) at 4°C for 30 min followed by flow cytometric analysis. Results were analyzed with the ModFitLT 3.2 software (Verity Software House, Inc., Topsham, ME, USA).

### Western blot

RPMI-8226 and OCI-MY5 cells were lysed on ice for 30 min in the lysate (100 mM Tris-HCl, pH 6.8, 4% SDS, 20% glycerol) and the supernatant was collected. Protein concentration was determined by BCA method (Beyotime Institute of Biotechnology, Haimen, China). Protein samples were separated by sodium dodecyl sulfate-polyacrylamide gel electrophoresis (SDS-PAGE). The protein of the desired molecular weight is then transferred to the nitrocellulose membrane and blocked with 5% non-fat dried milk at room temperature for 1 h. The membranes were incubated with primary antibodies overnight at 4°C. The next day, the membrane attached to corresponding protein was rinsed three times with PBST (1 × PBS-0.01% tween-20) for 10 min each time. The membrane was then probed with the corresponding secondary antibody for 1 hour at room temperature and protein bands were detected using the Odyssey two-color infrared laser imaging system (LI-COR Biosciences, Lincoln, USA).

### Quantification of pyruvate and lactate

Myeloma cells added to different concentrations of DCZ0801 were cultured in 24-well plates for 48 h, respectively. The sample was centrifuged at 142 x g for 5 min and then the supernatant was collected. The experiment was carried out using a commercial colorimetric kit (Nanjing Jiancheng Bioengineering Institute) according to the manufacturer's instructions. The absorbance values of pyruvate and lactate were determined by a microplate reader at wavelengths of 530 nm and 503 nm, respectively.

### Xenograft model

Five-week-old female BALB/C nude mice were purchased from the Shanghai Animal Experimental Center. The mice survived in an air-conditioned room at 24°C, humidity of 45%, and with 12 h light/dark cycle. The autoclaved food and water can be obtained without restriction and animal experiments were carried out after all mice had been acclimated for at least a week. Human OCI-MY5 cells (5 × 10^7^) in 100 μL serum-free culture medium were subcutaneously injected into the upper flank region of the nude mice. When the tumor was measurable, it was randomly assigned to the vehicle group and the DCZ0801 group. The control group was given 100 μL of placebo (10% DMSO, 30% HS-15 and 60% normal saline), while the DCZ0801 group was intraperitoneally injected DCZ0801 with 300 mg/kg. The size of the tumor and the weight of mice are measured daily and the volume of the tumor is calculated as (length × width^2^) × 0.5. At the end of the treatment, all mice were sacrificed by cervical dislocation. The tumor of the mouse was completely stripped from its skin and the vital organs such as the liver and kidney were removed and then fixed with 4% paraformaldehyde, followed by HE, TUNEL, Ki67 staining. All operations associated with animal experiments were approved by the Animal Care and Use Committee of Tongji University (Shanghai, China) and the institutional review board of the Shanghai Tenth People's Hospital (ID: SYXK 2018-0034).

### Statistical analysis

Data analysis was conducted using one-way ANOVA for multiple comparisons. All statistical analyses were performed using SPSS version 20.0 statistical analysis software (IBM Corp., Armonk, NY, USA). Statistically significant difference was set at P value of less than 0.05.

## Results

### DCZ0801 inhibits the survival and proliferation of multiple myeloma cells

**Figure 1:**
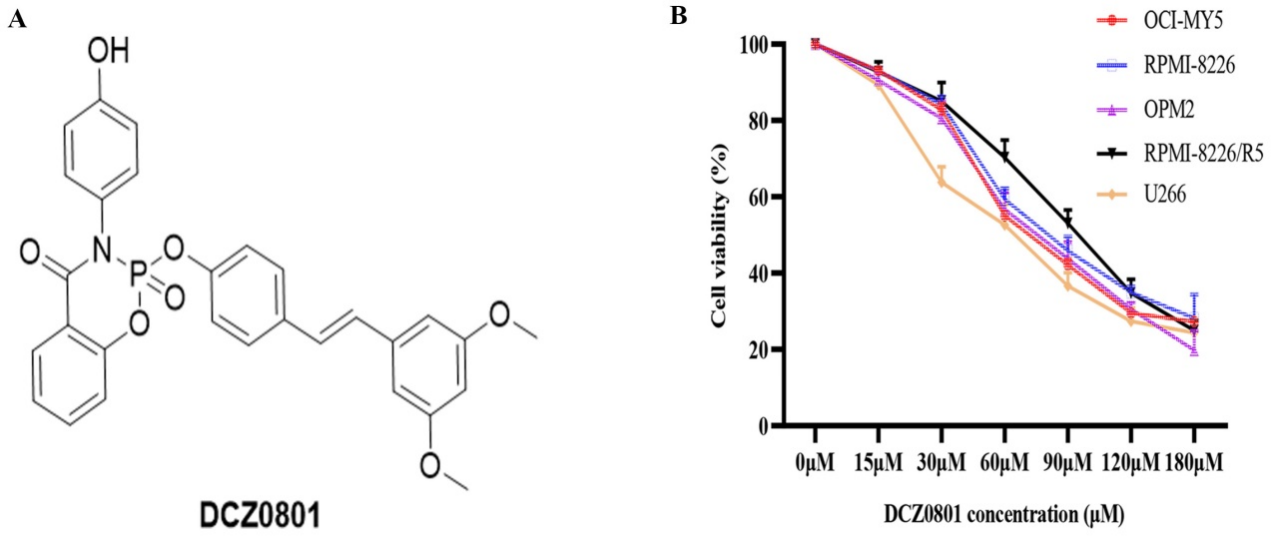
DCZ0801 inhibits proliferation of MM cells. **(A)** Molecular structure of DCZ0801. **(B)** MM cells were treated with different concentrations of DCZ0801 for 48 h. CCK-8 assay was used to detect cell viability.

DCZ0801 is a synthetic small molecule compound with a molecular weight of 529.48 (Figure 1A), mentioned in a previous article by our research group 35. To determine whether DCZ0801 has anti-myeloma activity, MM cells were exposed to different concentrations of DCZ0801 (15-180 μM) for 48 h and cell viability was then determined using the CCK-8 assay. As shown in Figure 1B, DCZ0801 could significantly decrease the survival of MM cells. The half maximal inhibitory concentration values of DCZ0801 for OCI-MY5, RPMI-8226, U266, OPM2 and RPMI-8226/R5 were 77.55 μM, 74.87 μM, 66.15 μM, 79.62 μM and 98.35 μM respectively. These results suggest that DCZ0801 can reduce cell viability and inhibit cell proliferation in a dose-dependent manner.

### DCZ0801 can induce apoptosis in MM cells

**Figure 2:**
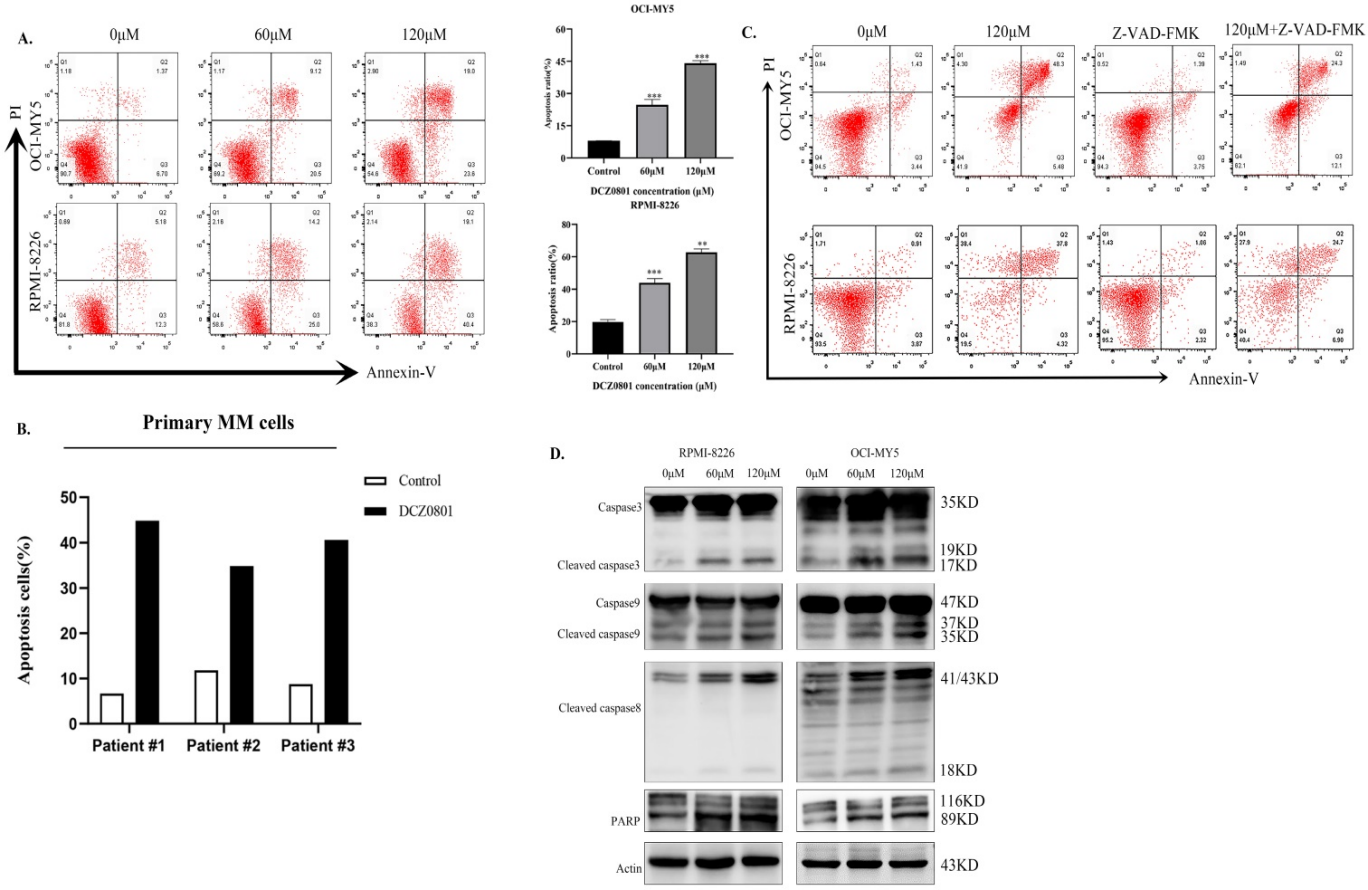
DCZ0801 can enhance apoptosis of MM cells. **(A)** MM cells treated with DCZ0801 were cultured for 48 h and detected by flow cytometry using Annexin V/PI staining. The average proportion of apoptosis from three independent experiments was presented in the column plot. Data are expressed as the means ± standard deviation (n = 3). *P < 0.05, **P < 0.01 and ***P < 0.001 compared with the 0 μM group. **(B)** Primary CD138^+^ plasma cells isolated from MM patients were treated with DCZ0801 (120 µM, 48 h) followed by apoptosis analysis. Column represents apoptosis rates of primary MM cells. Data represent mean ± SD from three separate experiments. **(C)** Cells were incubated with or without the pan-caspase inhibitor Z-VAD-FMK for 2 h, and then treated with 120 μM DCZ0801 for 48h. Treated cells were stained with Annexin V/PI and analyzed by FACS. **(D)** Western blot analyzed the expression levels of apoptosis-associated caspase family.

To investigate whether apoptosis in MM cells is associated with DCZ0801-induced cytotoxicity, myeloma cells were treated with DCZ0801 and analyzed by flow cytometry using Annexin-V/PI double staining. Consistent with the CCK-8 results, DCZ0801 significantly promoted apoptosis of MM cells in a dose-dependent manner (Figure 2A). We also observed the effect of DCZ0801 on myeloma primary cells and normal human peripheral blood mononuclear cells, and found that DCZ0801 can induce apoptosis in CD138^+^ myeloma cells isolated from the bone marrow mononuclear cells of myeloma patients (Figure 2B). On the contrary, a research showed that no significant cytotoxicity to normal human peripheral blood mononuclear cells was observed at the same dose 35. This shows that DCZ0801 has a selective killing effect between myeloma cells and normal cells. OCI-MY5 and RPMI-8226 cells treated with DCZ0801 were incubated with or without Z-VAD-FMK, a pan-caspase inhibitor. As shown in Figure 2C, the pan-caspase inhibitor reduced DCZ0801-induced apoptosis. Moreover western blot analysis showed an increase in the expression of apoptosis-associated proteins such as caspase-3, cleaved caspase-8, caspase-9, and PARP in OCI-MY5 and RPMI-8226 cells, suggesting the role of DCZ0801 in triggering these changes (Figure 2D). Based on the above findings, DCZ0801 was indicated to induce MM cell apoptosis through both the intrinsic and extrinsic caspase apoptotic pathways.

### DCZ0801 can induce myeloma cells arrest in the S phase

**Figure 3:**
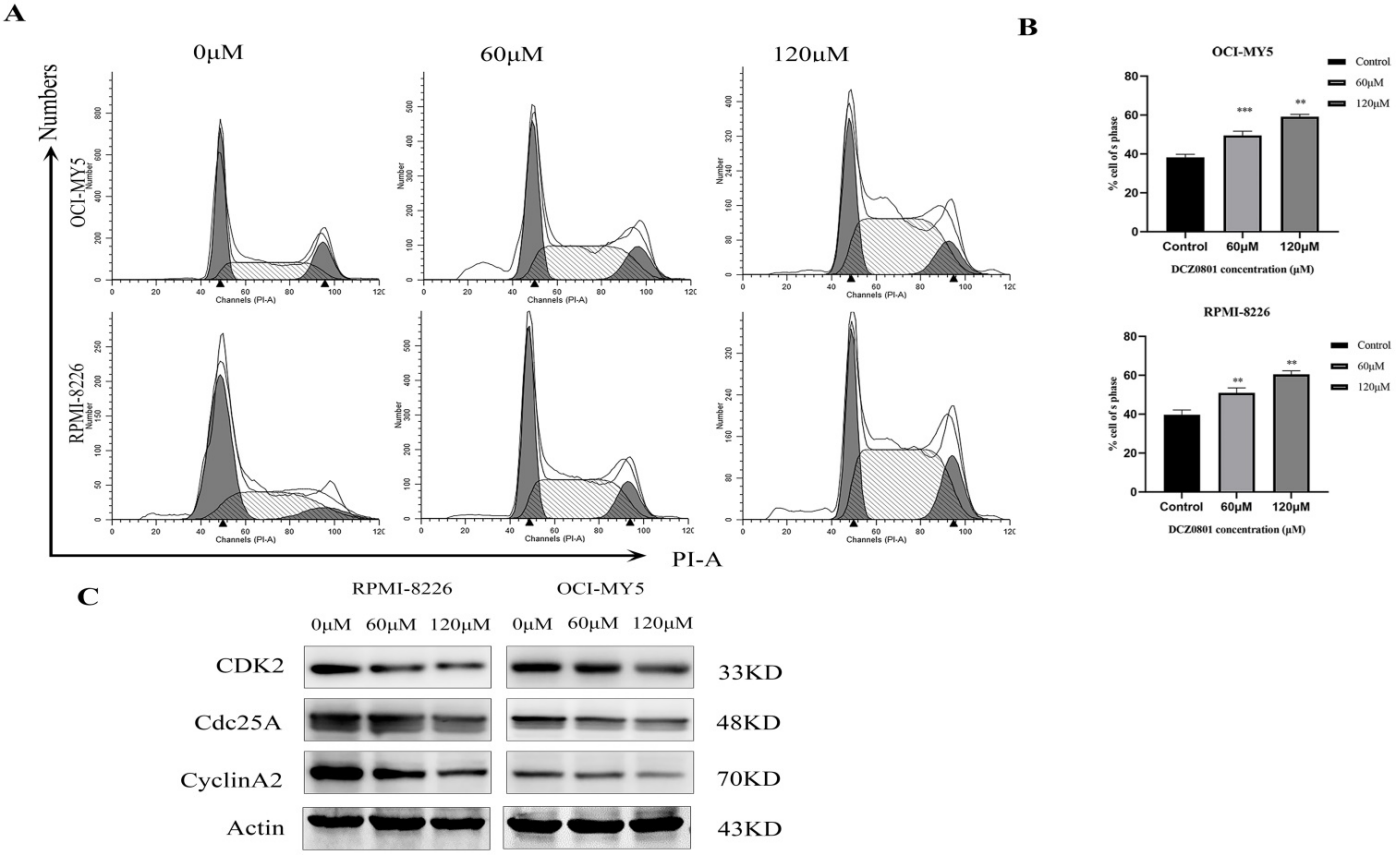
DCZ0801 induced S phase arrest in MM. **(A)** OCI-MY5 and RPMI-8226 were exposed to DCZ0801 (0, 60, 120 μM) for 24 h. Flow cytometry analyzed cell cycle by using PI staining. **(B)** % of cell in S phase from three groups of independent data was presented by Bar graph. **P < 0.01, ***P < 0.001. **(C)** Western Blot assessed the expression level of protein associated with S phase, with Actin used as an internal reference index.

We evaluated the cell cycle of OCI-MY5 and RPMI-8226 cells treated with DCZ0801 using flow cytometry. As shown in Figure 3A and 3B, DNA content analyses revealed that DCZ0801 treatment results in a dose-dependent increase in the cells in S phase and a decrease in the cells in the G0/G1 and G2/M phases. In addition, western blot analysis showed level of cell cycle regulatory proteins which accelerate process of S phase (Figure 3C). RPMI-8226 and OCI-MY5 cells treated with DCZ0801 for 24 h showed clearly downregulated levels of CDK2, Cyclin A2, and CDC25A in a dose-dependent manner. These data indicated that DCZ0801 could trigger S phase arrest in multiple myeloma cells by reducing the levels of cell cycle proteins, and thus blocking damaged DNA replication.

### DCZ0801 suppresses glycolysis in myeloma cells

**Figure 4:**
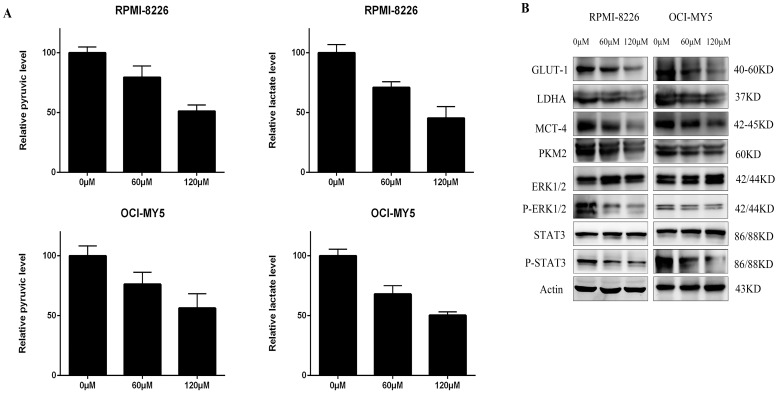
Glycolysis were repressed in MM. **(A)** After OCI-MY5 and RPMI-8226 cells were treated with DCZ0801 for 48 h, their absorbance was measured by a microplate reader using a lactate and pyruvate kit and the yield was calculated. **(B)** MM cells were treated with DCZ0801 for 48 h and then collected for western blot analysis. Protein expression of GLUT-1, LDHA, MCT4, PKM2, ERK1/2, p-ERK1/2, STAT3 and p-STAT3 were examined, with Actin acted as an internal control. GLUT-1, glucose transporter type 1; LDHA, lactate dehydrogenase A; PKM2, pyruvate kinase isozyme type M2; MCT4, monocarboxylate transporter 4.

Many studies have shown that the Warburg effect is prevalent in many tumors, such as multiple myeloma 36. In the present study, the levels of pyruvate and lactate in OCI-MY5 and RPMI-8226 cells were determined after 48 h treatment with DCZ0801, using the respective assay kit. As shown in Figure 4A, DCZ0801 reduced the yield of pyruvate and lactate produced by glycolysis in MM cells in a dose-dependent manner. Further, cell glycometabolism from protein levels was found to be suppressed by DCZ0801. Western blot analysis showed that DCZ0801 inhibited the protein expression levels of GLUT-1, LDHA, PKM2, and MCT4 in a dose-dependent manner. Moreover decreased glycolysis also reduced the phosphorylation of ERK1/2 and STAT3, thus affecting glucose uptake, lactate production and myeloma cell proliferation, without any changes in the total protein levels of ERK1/2 and STAT3 (Figure 4B). These results indicate that restraint of myeloma cell proliferation is mediated by DCZ0801 and may partly result from glycolysis suppression.

### DCZ0801 downregulates the Akt/mTOR signal pathway

**Figure 5:**
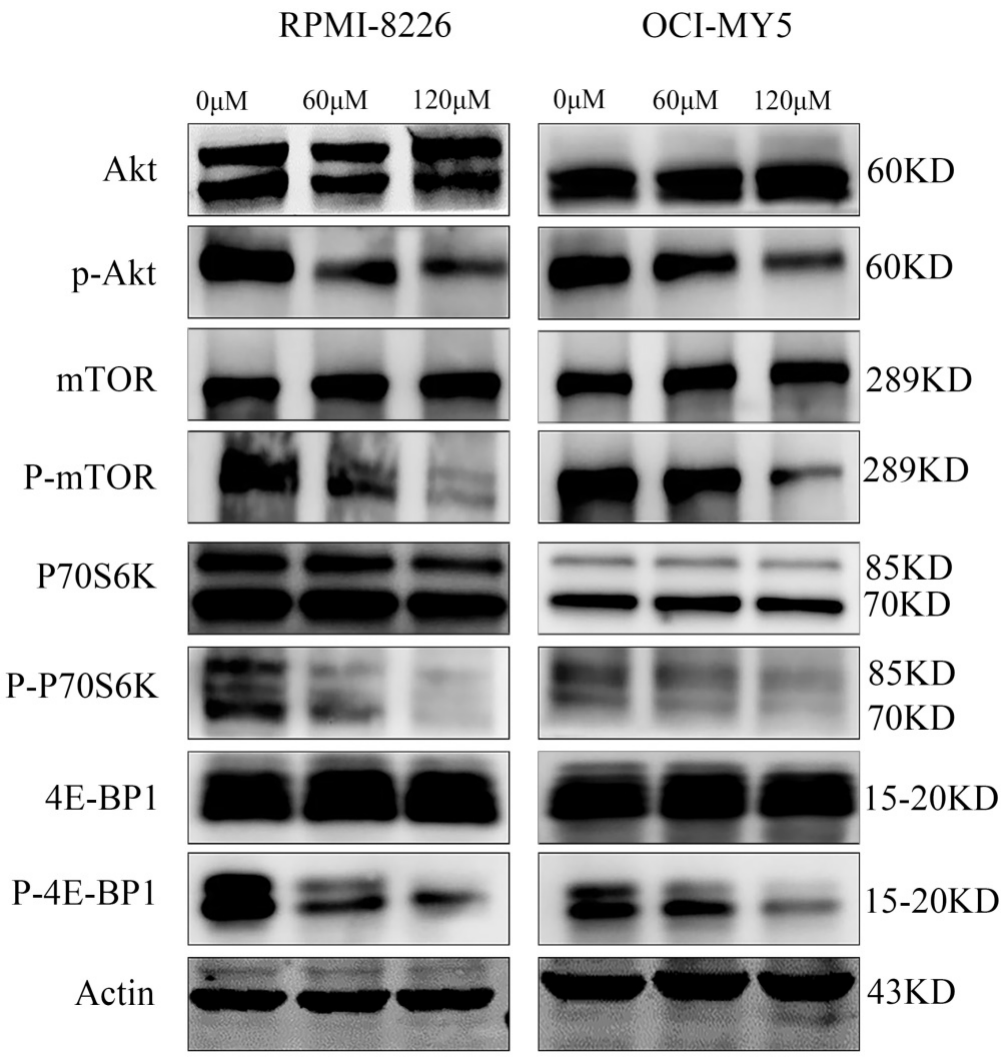
DCZ0801 regulated mTOR kinase activity. OCI-MY5 and RPMI-8226 were cultured with 60 μM and 120 μM DCZ0801 for 48 h. Western Blot analysis was used to evaluate the protein expression level of Akt, p-Akt, mTOR, p-mTOR, p70s6k, p-p70s6k, 4E-BP1, p-4E-BP1 and Actin.

To uncover the fundamental molecular events underlying the growth inhibition of myeloma cells induced by DCZ0801, we examined the expression levels of major signaling proteins associated with myeloma cell proliferation and survival using western blot analysis. Treatment of OCI-MY5 and RPMI-8226 cells with DCZ0801 (0, 60, 120 μM) clearly decreased the expression of phosphorylated Akt, mTOR, p70S6K, and 4E-BP1, with no significant changes in total Akt, mTOR, p70S6K and 4E-BP1 protein levels (Figure 5). Based on these results, DCZ0801 may block the Akt/mTOR signaling pathway by reducing pathway protein activation and phosphorylation.

### DCZ0801 inhibits tumor growth in a xenograft mouse model

**Figure 6:**
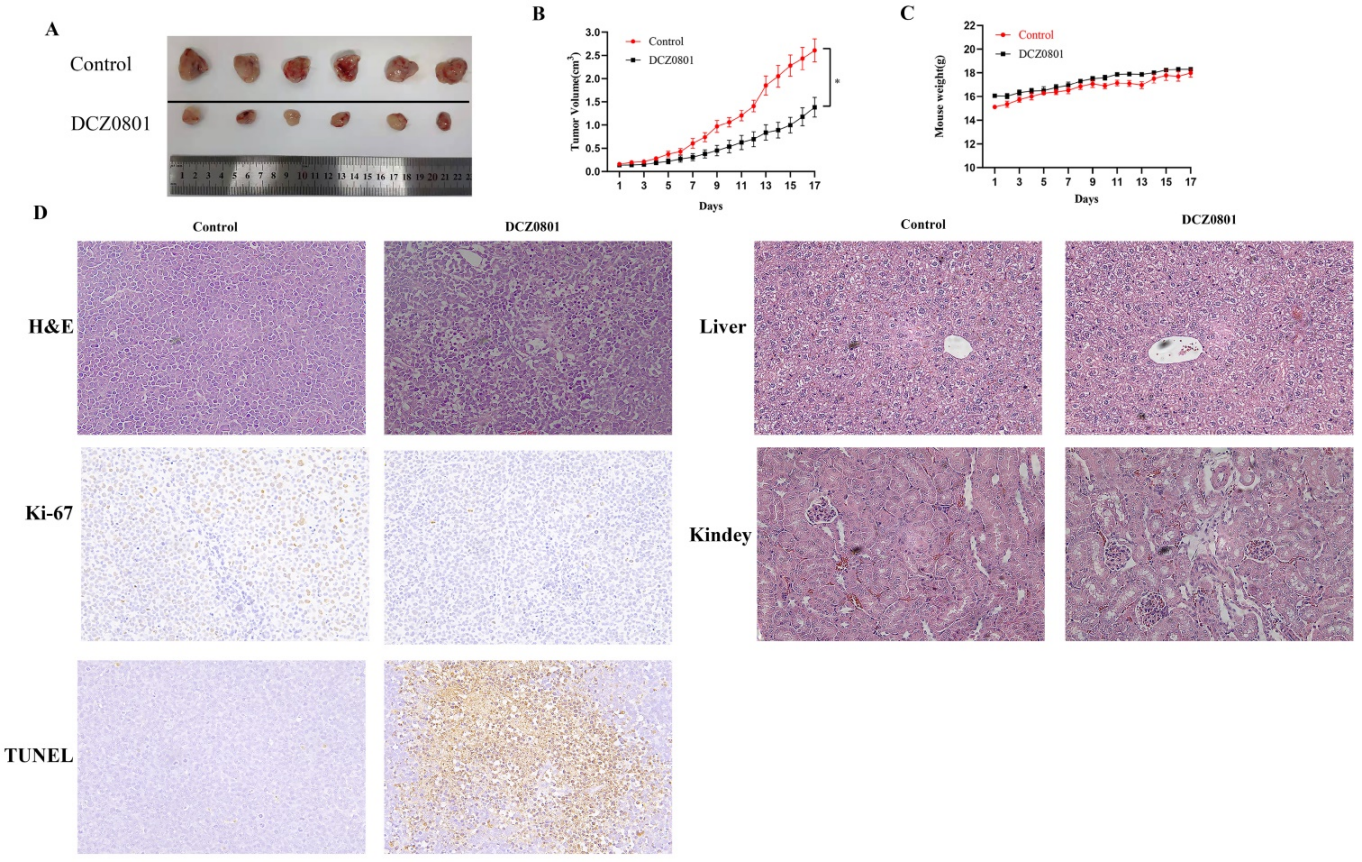
DCZ0801 has anti-tumor activity in vivo**. (A)** On Day 17, the tumors were obtained. **(B)** Nude mice bearing OCI-MY5 cells were subcutaneously injected placebo or DCZ0801 (300 mg/kg/day) daily for 17 days. The tumor volume was measured every day and tumor growth curve were completed (n = 6 mice/group, *P < 0.05). **(C)** The body weight of mice is obtained using an electronic scale for 17 days, and there were no significant difference between two groups. Data were shown as the mean ± SD. **(D)** H&E staining of the control and DCZ0801-treated tumor samples in the nude mice (original magnification: ×400); TUNEL and Ki67 staining of the control and DCZ0801-treated xenograft tumor tissues and H&E staining of control and DCZ0801-treated liver and kidney were obtained (original magnification: ×200).

To examine the effect of DCZ0801 *in vivo*, nude mice bearing subcutaneously inoculated OCI-MY5 cells were treated with daily intraperitoneal injection of DCZ0801 (300 mg/kg) or vehicle for 17 days. As shown in Figure 6A and 6B, DCZ0801 treatment decreased the tumor size compared with the control treatment. The tumor volume of the DCZ0801 treatment group was smaller than that in the control group. The body weights showed no significant differences between the DCZ0801 and control groups (Figure 6C). Further, examination of liver and kidney tissues by HE staining was carried out. The histomorphology of the liver and kidney was found to be significantly unchanged in the DCZ0801 treatment group compared with that in the control group (Figure 6D). These findings show that the side effects caused by DCZ0801 were well-tolerated and were not life-threatening in xenograft mice. HE staining revealed increased cell shrinkage and fragmentation in the tumor tissues of the DCZ0801 group compared to those of the control group. Moreover, an increase in TUNEL-positive cells and a decrease in Ki-67 expression was observed in the DCZ0801-treated group (Figure 6D), further confirming that DCZ0801 has potential anti-tumor effects *in vivo*.

## Discussion

With the development of new drugs for MM treatment, the response rates and overall survival continue to improve. However, MM is still an incurable and highly refractory disease 37. Therefore, seeking new and more effective therapies to target this disease has become extremely urgent. Osalmide targets the ribonucleotide reductase M2 protein, inhibits hepatitis B virus replication, and serves as a potential antiviral agent for chronic HBV infection 38. Pterostilbene, the 3,5-dimethoxy motif at the A-phenyl ring of resveratrol, is more lipophilic, and thus exhibits better bioavailability and therapeutic activity than resveratrol 39,40. Therefore, pterostilbene has received tremendous attention and has been reported to have powerful growth-inhibitory effects in several different types of cancer cells, notably hematological tumors 41. Based on these results, we synthesized the novel compound composed of osalmide and pterostilbene named DCZ0801,and explored the effects and relevant mechanisms of DCZ0801 on MM in this study.

We found that MM cells treated with DCZ0801 show inhibition of cell survival and growth via increased caspase-dependent apoptosis and induction of cell cycle arrest. Accurate cell cycle progression regulates the normal cell division, which is a series of steps achieved by specific cyclins that act in association with cyclin-dependent kinases. The activation CDK2-CyclinA2 complexes by dephosphorylation of CDC25A can promote S phase progression and DNA duplication. Therefore when CDC25A was inhibited by DNA damage checkpoint kinase activation, cell cycle would get blocked in S phase 42,43. In this study, treatment of myeloma cells with DCZ0801 down-regulates the level of CDK2, Cyclin A2, and CDC 25A, which arrests the cell cycle in the S phase. Apoptosis is a programmed cell death process whose dysfunction is associated with uncontrolled cell proliferation, aggressiveness, metastasis, and drug resistance to anti-cancer therapies 44,45. Caspases play an important role in apoptosis. Activation of the initiator caspase 8 and caspase 9 in the extrinsic and intrinsic cell death pathways activates the executor caspase 3, eventually leading to cell death 46. Flow cytometry analysis showed that cell apoptosis induced by DCZ0801 increases in a dose-dependent manner and was blocked by the pan-caspase inhibitor, Z-VAD-FMK. Moreover, DCZ0801 treatment increased the expression levels of caspase 3, cleaved caspase 8, caspase 9, and PARP proteins. These findings indicate that DCZ0801 stimulates apoptosis via the extrinsic and intrinsic caspase-apoptosis pathways.

Energy metabolism reprogramming by which tumor cells attain growth and proliferation advantages is well-known as an emerging hallmark of cancer 47,48. Cancer cells frequently exhibit high rates of glycolytic flux in comparison to normal cells, in that, glycolytic intermediates facilitate biosynthesis of nucleotides, amino acids, and lipids that are essential to tumor cell metabolism 49. In this study, cells treated with DCZ0801 showed a decrease in the content of pyruvate and lactate, with reduced levels of glycolysis enzymes (GLUT1, LDHA, MCT4, PKM2), indicating that DCZ0801 can obstruct the enhanced glycolysis in MM cells. High aerobic glycolysis enhances the transcriptional activation of STAT3 and promotes the phosphorylation and activation of ERK1/2 19. Treatment of MM cells with DCZ0801 markedly decreased the ERK1/2 and STAT3 phosphorylation as seen by western blot analysis. Therefore, we concluded that DCZ0801 inhibits increased aerobic glycolysis to decrease the generation of glycolytic intermediates, leading to tumor cell growth and proliferation retardation.

mTOR is a serine/threonine protein kinase, whose aberrant activation is very prevalent in malignant cells, contributing to tumor formation and progression 50. Upregulation of mTOR is required for increased GLUT1 expression and glucose consumption. mTOR renders the metabolic program switch to aerobic glycolysis in tumor cells by activating HIF1α, a positive regulator of many glycolytic genes 51,52. Therefore, in oncogenic signaling, mTOR directly phosphorylates downstream effectors such as 4E-BP1 and S6K1, which in turn translates HIF1α to improve glycolysis, finally promoting the survival of tumor cells. In this study, the western blot results demonstrated that DCZ0801 can reduce the phosphorylation of Akt, mTOR, p70S6K and 4E-BP1. Therefore, we concluded that DCZ0801 suppresses aerobic glycolysis by down-regulating Akt/mTOR pathway activation, which leads to inhibition of cell proliferation.

To investigate whether DCZ0801 has a proliferation inhibitory effect *in vivo*, we established a xenograft mouse model. After intraperitoneal injection of 300 mg/kg DCZ0801 for 17 days, DCZ0801 was found to significantly inhibit tumor growth without any major changes in mouse weight. Thus, consistent with the *in vitro* results, DCZ0801 exerts anti-tumor activities *in vivo* without lethal toxicity.

In conclusion, our study suggests that DCZ0801 has potential anti-tumor activity both *in vitro* and *in vivo*. *In vitro*, exposure to DCZ0801 induces caspase-dependent apoptosis and cell-cycle arrest to counter tumor proliferation in MM. It can also suppress the activation of glycolysis resulting from inhibition of Akt/mTOR pathway. In addition, DCZ0801 inhibited tumor growth in a xenograft model *in vivo*. Overall, the new natural compound, DCZ0801, which has a different mechanism of action compared to the current anti-MM drugs, could serve as a potential candidate for the treatment of MM.

## Abbreviations

MM: multiple myeloma
CAR-T cell: Chimeric Antigen Receptor T-Cell
CCK-8: cell counting kit-8
PBMCs: peripheral blood mononuclear cells
GLUT-1: glucose transporter type 1
LDHA: lactate dehydrogenase A
PKM2: pyruvate kinase isozyme type M2
MCT4: monocarboxylate transporter 4
PI: propidium iodide
HE: hematoxylin eosin
TUNEL: terminal deoxynucleotidy1 transferase dUTP nick end-labeling
